# Visualizing wax ester fermentation in single *Euglena gracilis* cells by Raman microspectroscopy and multivariate curve resolution analysis

**DOI:** 10.1186/s13068-019-1471-2

**Published:** 2019-05-22

**Authors:** Keita Iwasaki, Asuka Kaneko, Yuji Tanaka, Takahiro Ishikawa, Hemanth Noothalapati, Tatsuyuki Yamamoto

**Affiliations:** 1 The United Graduate School of Agricultural Sciences, Tottori University, Tottori, 680-8550 Japan; 2 Faculty of Life and Environmental Science, Shimane University, Matsue, 690-8504 Japan; 3 Core Research for Evolutional Science and Technology (CREST), Japan Science and Technology Agency (JST), Kawaguchi, 332-0012 Japan; 4 Raman Project Center for Medical and Biological Applications, Shimane University, Matsue, 690-8504 Japan

**Keywords:** *Euglena*, Wax ester, Myristyl myristate, Biofuel, Jet fuel, Raman microspectroscopy, Multivariate curve resolution, Label-free imaging, Molecular imaging

## Abstract

**Background:**

Global demand for energy is on the rise at a time when limited natural resources are fast depleting. To address this issue, microalgal biofuels are being recommended as a renewable and eco-friendly substitute for fossil fuels. *Euglena gracilis* is one such candidate that has received special interest due to their ability to synthesize wax esters that serve as precursors for production of drop-in jet fuel. However, to realize economic viability and achieve industrial-scale production, development of novel methods to characterize algal cells, evaluate its culture conditions, and construct appropriate genetically modified strains is necessary. Here, we report a Raman microspectroscopy-based method to visualize important metabolites such as paramylon and ester during wax ester fermentation in single *Euglena gracilis* cells in a label-free manner.

**Results:**

We measured Raman spectra to obtain intracellular biomolecular information in *Euglena* under anaerobic condition. First, by univariate approach, we identified Raman markers corresponding to paramylon/esters and constructed their time-lapse chemical images. However, univariate analysis is severely limited in its ability to obtain detailed information as several molecules can contribute to a Raman band. Therefore, we further employed multivariate curve resolution analysis to obtain chain length-specific information and their abundance images of the produced esters. Accumulated esters in *Euglena* were particularly identified to be myristyl myristate (C28), a wax ester candidate suitable to prepare drop-in jet fuel. Interestingly, we found accumulation of two different forms of myristyl myristate for the first time in *Euglena* through our exploratory multivariate analysis.

**Conclusions:**

We succeeded in visualizing molecular-specific information in *Euglena* during wax ester fermentation by Raman microspectroscopy. It is obvious from our results that simple univariate approach is insufficient and that multivariate curve resolution analysis is crucial to extract hidden information from Raman spectra. Even though we have not measured any mutants in this study, our approach is directly applicable to other systems and is expected to deepen the knowledge on lipid metabolism in microalgae, which eventually leads to new strategies that will help to enhance biofuel production efficiency in the future.

## Background

Fossil fuels contribute to two-thirds of the global energy demand out of which oils contribute 33% [1, 2]. In an age of increasing population growth, overconsumption, and depleting oil supplies, continued use of petroleum sourced fuels is both unsustainable and damaging to environment with long-standing negative impacts on public health and global climate [3, 4]. Therefore, there is an urgent need to find suitable renewable energy sources. Microalgal biofuels are currently the most favored substitute for liquid fossil fuels than other nontoxic, eco-friendly alternatives such as plant or animal biomass derived energy. Microalgae offers several advantages: (1) easy and quick growth under various conditions, (2) does not compete for arable land and water with edible crops, and (3) provides carbon neutral renewable energy by converting CO_2_ to useful products such as fatty acids, alcohols, and neutral lipids. Many algae generally produce substantial amounts of triacylglycerol of medium-chain fatty acids such as palmitic (C16:0) and stearic (C18:0) acids, sometimes up to 70% of its dry weight [5, 6].

One such microalgae that has received considerable attention in the past few decades as a biotechnological tool to produce drop-in jet fuel is *Euglena gracilis*, a photosynthetic unicellular flagellate eukaryote. *Euglena,* being a mixotroph, feeds as an autotroph in the presence of sunlight to produce sugars through photosynthesis, while survives as a heterotroph taking in dissolved organic compounds as nutrition under dark conditions. One of the main reasons for its attraction is because of its ability to produce wax esters, chiefly myristyl myristate (MM). MM is made up of myristic (C14:0) acid and myristyl alcohol (C14:0), each of which can individually be utilized for jet fuel because of their low freezing point/high cetane number compared to other medium-chain fatty acids [7]. Typically, *Euglena* cells accumulate storage polysaccharide called paramylon granules, a β-1,3-glucan under aerobic conditions. However, such stored paramylon is broken down to glucose and further converted to wax esters when put under anaerobic conditions. Since the anaerobic cells gain subtle levels of ATP during the process, the phenomenon is called “wax ester fermentation” [8].

Though *Euglena* cells have huge potential and can serve as tiny factories for biofuel production, inherent problem associated with large-scale culturing is the slow growth rate of algal strains with high oil content [8, 9]. It appears that the synthesis and storage of wax esters as cytosolic lipid particles is *Euglena’s* defense mechanism to cope with stress [5]. Therefore, much effort has been put to genetically engineer or optimize culturing conditions of algae for enhanced biofuel production [8, 10–13]. To evaluate any constructed algal strain or the choice of culture conditions, polysaccharide/lipid profiles must be characterized. The conventional quantification methods employ labor intensive, time consuming, and destructive chemical extraction procedures followed by expensive mass spectrometric measurements, thereby limiting scientific progress.

Therefore, we set out to develop a Raman spectroscopy (RS)-based molecular imaging method to characterize various metabolites in *Euglena* in a simple and straightforward manner. Raman spectrum, which is also called a molecular fingerprint, provides wealth of chemical information with high specificity. Combining RS with a microscope endows subcellular resolution. Moreover, it is a rapid, non-destructive, live cell compatible technique that requires no additional dye probes or extensive sample preparation for molecular imaging. Previously, metabolic heterogeneity of live *Euglena* was studied in real time by stimulated Raman scattering. However, only the heavily crowded C–H-stretching region could be analyzed [14]. Spontaneous RS has also proved to be useful in studying enhanced lipid production in yeasts [15]. In this work, we performed space- and time-resolved Raman imaging of single living *Euglena* cells under anaerobic conditions and analyzed fingerprint region rich in molecular and structural information to identify/visualize paramylon and products of wax ester fermentation.

We identified Raman spectral markers for β-1,3-glucan/esters and constructed their intracellular distribution images by simple univariate approach. To obtain carbon chain length-specific information of lipids and further probe any other unknown components, we employed multivariate curve resolution (MCR) analysis and succeeded in identifying MM (C28), a major product of wax ester fermentation which is an ideal raw material for a drop-in bio jet fuel.

## Results and discussion

### Raman microspectroscopy and imaging of single *Euglena gracilis* cells

**Fig. 1:**
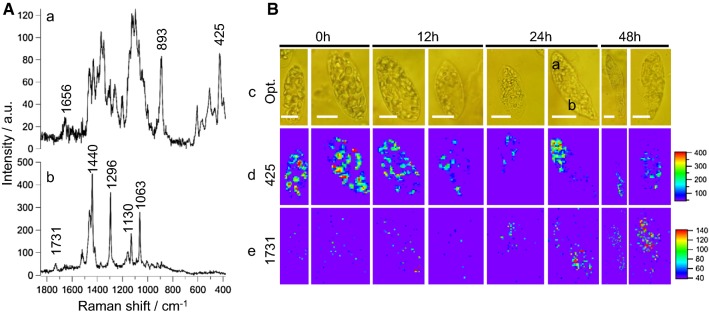
Raman microspectroscopy and imaging of single *Euglena gracilis* cells under anaerobic condition. **A** Space-resolved Raman spectra measured at polysaccharide-rich region (a), and ester-rich region (b) from a cell at 24 h. **B** Optical images of single *E. gracilis* cells (c), time-resolved univariate Raman images of polysaccharides (d), and esters (e). Scale bar in each optical image measures 10 μm and measured points are indicated using alphabets

To understand wax ester fermentation in *Euglena* at the molecular level, we measured space- and time-resolved Raman spectra and images of single cells grown under anaerobic conditions (Fig. 1). As mentioned earlier, stored polysaccharides in *Euglena* are converted to wax esters. Therefore, to identify and discuss Raman spectral markers during wax ester fermentation, two most relevant space-resolved Raman spectra from a *Euglena cell* are presented in Fig. 1A.

**Scheme 1:**
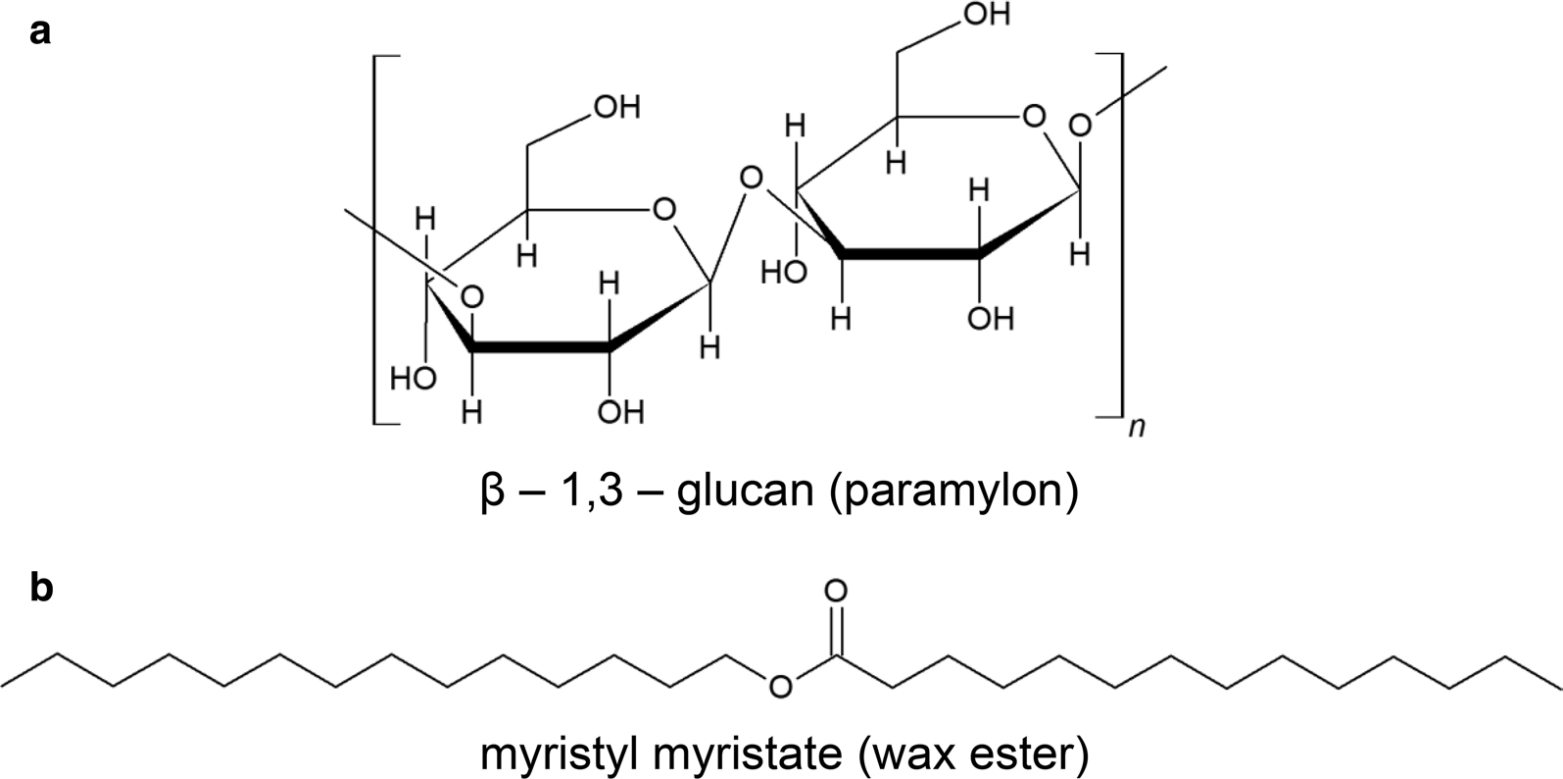
Molecular structures. **a** β-1,3-glucan (paramylon) and **b** myristyl myristate (wax ester)

Spectrum at point a (Fig. 1A-a) had COO^−^ asymmetric stretching at 1656 cm^−1^, COO^−^ symmetric stretch, and C–H deformation modes between 1500 and 1200 cm^−1^, C–C and C–O stretch modes of pyranose rings between 1150 and 1050 cm^−1^, and C–C–C ring deformation mode at 425 cm^−1^ indicating polysaccharide-rich region. In addition, we observed a band at 893 cm^−1^, a region which is sensitive to glycosidic linkages. In fact, Raman spectroscopic studies on series of carbohydrate monomers have revealed C–H equatorial bending vibration of β-anomer between 905 and 885 cm^−1^ [16–18]. We can safely assume that the observed polysaccharide spectrum may particularly be rich in paramylon, a β-glucan (Scheme 1a). However, actual comparison with pure β-glucan is necessary. Major features in Raman spectrum measured at point b (Fig. 1A-b) include C=O stretch of ester linkage at 1731 cm^−1^, C–H-bending vibrations of the aliphatic chain at 1440 cm^−1^, in-plane CH_2_ twist at 1296 cm^−1^, and C–C stretch between 1150 and 1050 cm^−1^. It is important to note that the absence of any band in the C=C stretch region around 1650 cm^−1^ clearly indicates that this strain only accumulates esters containing saturated hydrocarbon chains (Scheme 1b). It is then straightforward to choose 425 cm^−1^ and 1731 cm^−1^ bands to be markers of paramylon and wax esters, respectively.

To visualize dynamic intracellular distributions of these components, we performed time-resolved Raman imaging experiment of single *Euglena* cells at 0 h, 12 h, 24 h, and 48 h under anaerobic conditions. Two representative cells at each time are presented in Fig. 1B. It is apparent from univariate Raman images that cells at 0 h (pre-grown under aerobic conditions) have accumulated polysaccharides, while ester content is negligible. As the culture time progresses, stored polysaccharide content decreases slowly, while wax esters start accumulating, especially from 24 h. This is a clear indication of wax ester fermentation in *Euglena*.

### Identification of carbon chain lengths in wax esters

**Fig. 2:**
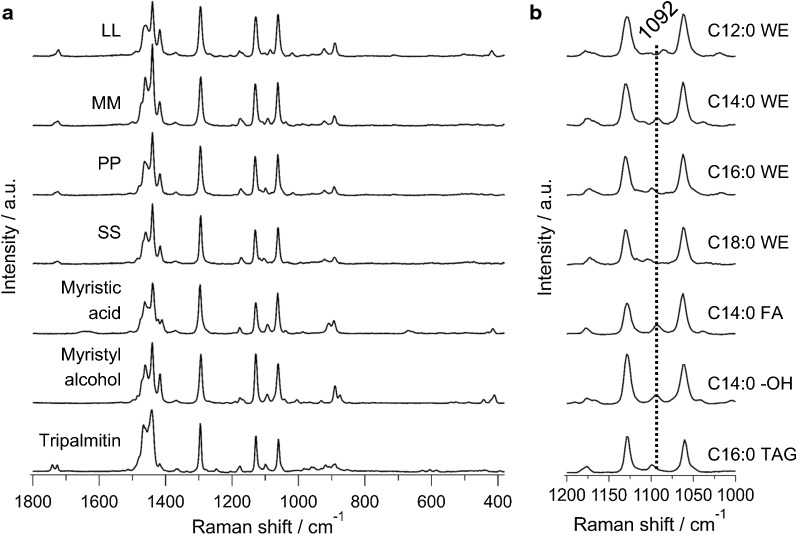
Comparison of Raman spectra of standard wax esters, lipid, and alcohol. **a** Fingerprint region (1800–800 cm^−1^) of lauryl laurate (LL), myristyl myristate (MM), palmityl palmitate (PP), stearyl stearate (SS), myristic acid, myristyl alcohol, and tripalmitin. **b** Enlarged view of the 1200–1000 cm^−1^ region containing C–C stretch information, which is useful for chain length analysis. Corresponding carbon chain lengths for each compound is indicated using common notation. Fluorescence background was subtracted using a polynomial baseline and all spectra were normalized to 1296 cm^−1^ band

Though we were able to visualize the fatty acid biosynthetic machinery at work, there is no information on the nature of wax esters produced. Because, the C=O stretch of ester linkage (1731 cm^−1^) used for molecular imaging does not indicate carbon chain lengths in compounds containing > 12 carbons [19], which is quite important in the context of its application for biofuel production. Therefore, to characterize the chain length of wax esters in detail within single *Euglena* cells, we set out to identify Raman markers that are sensitive to carbon chains. To achieve this, we measured series of standard wax esters with different chain lengths together with myristic acid and myristyl alcohol, precursors of MM which is a promising candidate for drop-in jet fuel (Fig. 2).

It is known that the position of C=O-stretching band in fatty acid methyl ester depends on the chain lengths. However, it is useful only for oils containing < 12 carbon atoms and the change in band position is minimal for fatty acids > 12 carbons [19]. Although the overall spectral pattern looked very similar (Fig. 2a), careful screening of C–C-stretching region revealed significant difference that can be attributed to chain lengths (Fig. 2b). Raman bands at 1130 cm^−1^ and 1063 cm^−1^ have been assigned to in-phase and out-of-phase skeletal C–C-stretching vibrations, respectively, for all-*trans* chain conformation. The band in between these two is a superposition of all-*trans* C–C stretch with a single *gauche* defect and C–C stretching of *gauche* conformation which is indicative of *gauche* isomer formation [20–23]. Its position has been found to be sensitive to carbon chain lengths and we observed a systematic shift to higher wavenumber with increasing carbon number (Table 1).

**Table 1:** Carbon chain length dependence of *gauche* conformation sensitive C–C-stretching band in saturated chains

Compound	Chain length	Band position^a^ (cm^−1^)
Lauryl laurate	12:0/12:0	1084.7 ± 0.9
Myristyl myristate	14:0/14:0	1092.2 ± 0.8
Palmityl palmitate	16:0/16:0	1099.1 ± 0.8
Stearyl stearate	18:0/18:0	1103.7 ± 0.9
Myristic acid	14:0	1092.6 ± 0.4
Myristyl alcohol	14:0	1094.1 ± 0.3
Tripalmitin	16:0/16:0/16:0	1098.6 ± 0.7

^a^Gaussian fitting was used to determine band positions and fitting errors are included

MM and both its precursors which contain C14:0 show Raman band close to 1092 cm^−1^ while others are shifted in either direction. Even though we succeeded in identifying chain length-specific Raman spectral markers, we must keep in mind that the difference in band position is quite small and that the measured samples were all pure compounds in solid state. This indicator has been shown to fail if the lipids are in liquid state [19].

### Extracting pure biomolecular information using MCR analysis

In the present context, Euglena cells contain heterogeneous distributions of many different biomolecules with varying phases and Raman spectrum measured at any given point in the cell is a mixture of all components. For example, C–C-stretching region of Raman spectrum is quite crowded with overlapping contributions not only from lipids or esters but also from other intracellular biomolecules such as protein, nucleic acids, polysaccharides, etc. Therefore, simple univariate approach is not suitable for such complex biological samples, especially to predict chain lengths. In fact, if we take a closer look into the space-resolved spectrum from ester-rich region between 1150–1050 cm^−1^ (Fig. 1A-b), it is hard to find any C–C gauche band. However, a broad and an intense band can be observed in the same region from polysaccharide-rich Raman spectrum (Fig. 1A-a) indicating the complexity involved. Therefore, we applied MCR analysis to extract pure biomolecular information and to visualize intracellular abundance of each component in a straightforward manner.

**Fig. 3:**
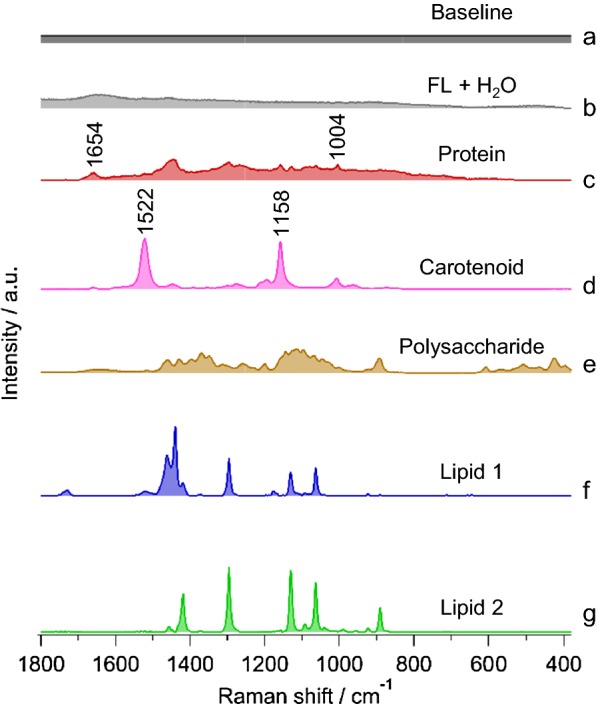
Results of MCR analysis assuming seven components. (a) Baseline, (b) fluorescence background (FL) and water, (c) protein, (d) carotenoid, (e) polysaccharide, and (f, g) lipids 1 and 2, respectively

Results of seven components MCR model is given in Fig. 3 in which a straight baseline was intentionally included to eliminate varying offset. Other six components were automatically extracted. Let us look into the assignment of each in detail. Figure 3b includes O–H-bending vibration of water around ~ 1600 cm^−1^ and an overall broad fluorescence background. Raman spectrum in Fig. 3c includes phenylalanine ring breathing mode at 1004 cm^−1^ and amide I band at 1660 cm^−1^, indicating proteins. Next component (Fig. 3d) contains intense bands at 1522 cm^−1^ and 1158 cm^−1^ which represent stretching modes of C=C and C–C of polyene chain in carotenoids, respectively. It is important to note that protein and carotenoid spectra were obtained as a natural consequence of MCR analysis without any a priori knowledge of their presence and, thus, could be very useful in exploratory analysis. Spectrum in Fig. 3e can be assigned to polysaccharide. Unexpectedly, we extracted two lipid components, named lipid 1 and 2, as shown in Fig. 3f, g, respectively.

### Comparison of MCR-extracted components with pure standards

**Fig. 4:**
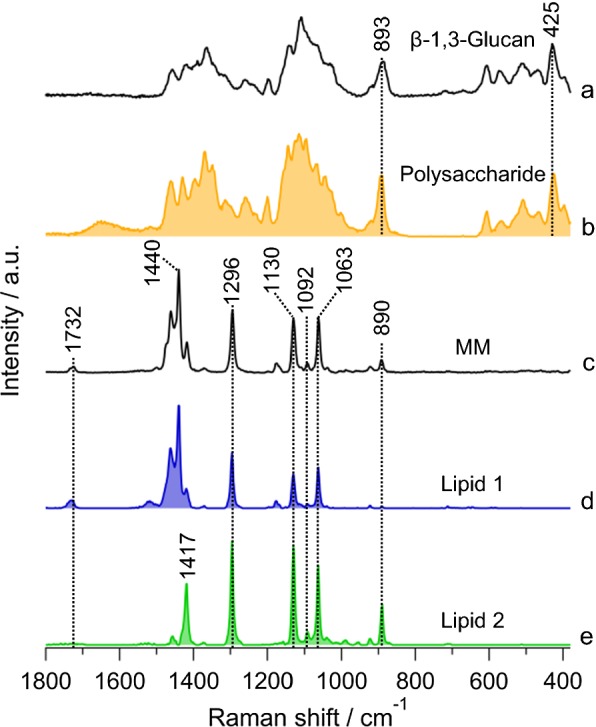
Comparison of MCR spectral components with pure standards. (a) β-1,3-glucan, (b) MCR-extracted polysaccharide (same as Fig. 3e), (c) myristyl myristate (MM), and (d, e) MCR lipids 1 and 2, respectively (same as Fig. 3f, g)

To understand the origin of polysaccharide and lipids from MCR analysis more specifically, we compared them with series of pure chemical standards of wax esters and their precursors. After screening, comparison with expected compounds such as β-1,3-glucan and MM is shown in Fig. 4. This is mainly because *Euglena* is known to store appreciable amounts of paramylon (a β-1,3-glucan) as energy reserves under aerobic conditions which are almost converted exclusively to wax esters containing saturated carbon chains. Gas chromatographic analysis showed esters with C28 to be the major component along with minor contributions from other even numbered esters in C24–C32 range [24]. Indeed, MCR-extracted polysaccharide component matches very well with β-1,3-glucan and can unambiguously be assigned to paramylon in *Euglena*. It is intriguing that two seemingly similar lipid components were extracted separately in MCR analysis (Fig. 4d, e). Lipid 1 with bands at 1732 cm^−1^, 1440 cm^−1^, 1296 cm^−1^, 1130 cm^−1^, 1092 cm^−1^, and 1063 cm^−1^ matches quite well with MM and can be assigned to C28 ester containing two saturated C14 chains. A closer look into lipid 2 reveals the absence of 1732 cm^−1^ and C–H-bending vibrations at 1440 cm^−1^, while 1417 cm^−1^ and 890 cm^−1^ are more pronounced. Absence of C=O stretch band of ester raises the question whether lipid 2 is really a lipid/ester. However, the presence of 1092 cm^−1^ along with other C–C-stretching vibrational bands indicates C14 carbon chain, indirectly suggesting that it could either be myristic acid or myristyl alcohol. However, it does not correspond well with neither as expected, especially in C=O stretch and C–H deformation region, essentially leaving the spectrum unassigned.

### MCR analysis of Raman images of standard myristyl myristate

**Fig. 5:**
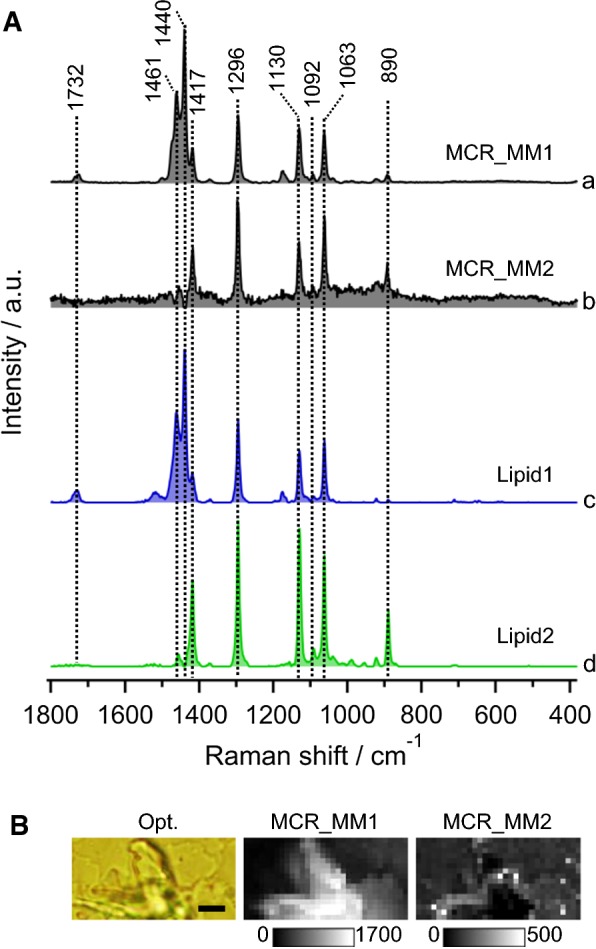
Results of MCR analysis of pure myristyl myristate. **A** Comparison of two MCR-extracted spectra from (a, b) pure myristyl myristate solid (MCR_MM1 and 2), and (c, d) *Euglena* (lipid 1 and 2). **B** Molecular distribution images of extracted components in pure myristyl myristate after MCR analysis. Corresponding optical image is included (Opt.). Scale bar measures 5 μm

Since lipid 2 extracted from *Euglena* with 1092 cm^−1^ band does not match either with wax ester or their precursors, we performed Raman imaging on pure MM solid film (obtained after drying 10 mg/ml MM in hexane) and carried out detailed MCR analysis (Fig. 5). A two-component MCR model constructed from data of pure MM showed surprising results. The two spectra were, indeed, identical to the two lipid components obtained from the MCR analysis of living *Euglena* cells, i.e., Fig. 5A-a (MCR_MM1) and Fig. 5A-c (lipid 1) were identical and both correspond well to averaged MM spectrum measured earlier (Fig. 4c). Spectral profile of second component (MCR_MM2), in which bands at 1732 cm^−1^ and 1440 cm^−1^ were missing, was identical to ‘lipid 2’ from *Euglena* cells, indicating its origin to MM. Only plausible explanation is the presence of crystal polymorphs (several crystalline structures with the same chemical composition).

It is known that long-chain esters/triglycerides exist in three major polymorphic forms, namely *α*, *β*′, and *β*. Their stability varies in the order *β* > *β*′ > *α*. While the subcell structure of α form is hexagonal with no ordered arrangement of chain planes (*H*), *β*′ is orthorhombic with every second chain being perpendicular to the rest (*O*_⊥_) and *β* is triclinic with all chain planes parallel (*T*_//_). In a Raman spectrum, C–H deformation modes between 1500 and 1400 cm^−1^ are sensitive to crystal structure. First set of spectra (MCR_MM1 and lipid 1) in which three defined bands at 1461 cm^−1^, 1440 cm^−1^, and 1417 cm^−1^ were observed corresponding to *β*′ polymorph. In fact, 1417 cm^−1^ band is associated with splitting of the Raman active methylene scissoring mode in *β*′ form [25–27]. In the second set, MCR_MM2 and lipid 2, intense bands at 1417 cm^−1^, 1296 cm^−1^, and 1130 cm^−1^ that are characteristics of all-*trans* conformation of carbon chains in crystalline domains were observed [28]. In addition, 890 cm^−1^ band corresponding to terminal C–C-stretching vibration was also prominent. However, it is interesting to note that C=O-stretching (1732 cm^−1^) and C–H-bending (1440 cm^−1^) vibrations in both spectra were absent. Since intensities of Raman bands depend on both crystal orientation and incident polarization, it is possible that these bands are weak in this particular sample due to crystal orientation. However, it may also be due to the presence of two polymorphs of MM in *Euglena* cells. This may have serious implications as physical properties like molecular packing and freezing point, which are crucial for MM’s efficient storage and eventual application as a bio jet fuel, will be different for different polymorphs. Polarized-Raman spectroscopic measurements should be performed to obtain further insights to make clear distinction between polymorphs.

We then constructed molecular distribution images of MCR-extracted components which revealed heterogeneous pattern without much resemblance to each other (Fig. 5B). This result further confirms the presence of two different forms in the standard MM sample.

### Time-resolved MCR component images of *Euglena* cells

**Fig. 6:**
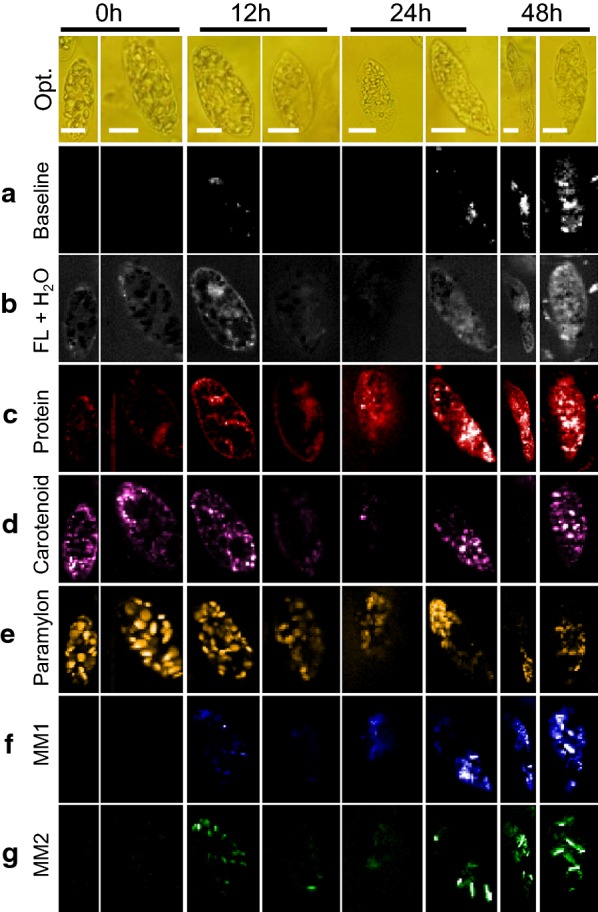
Raman images constructed from MCR analysis. (a) Baseline, (b) fluorescence background and water, (c) protein, (d) carotenoid, (e) paramylon, and (f, g) myristyl myristate 1 and 2, respectively. Corresponding optical images are included (Opt.). Scale bar measures 10 μm. Color scale in Raman images indicates molecular abundance

Once the assignment of all MCR-extracted spectral components was accomplished, we constructed time-resolved Raman images to visualize intracellular biomolecular distribution (Fig. 6). First, let us look into baseline (Fig. 6a). Although there is no difference at early culture times, significant increase in localized areas was observed in cells from 24 h. On the other hand, varying degrees of fluorescence background could be observed in cells at any given time (Fig. 6b). Protein synthesis seems to be active as its intracellular abundance increases and gets more or less evenly distributed throughout the cells as culture time progresses (Fig. 6c). However, irrespective of time, carotenoids were randomly distributed indicating cellular individuality (Fig. 6d). Details on wax ester fermentation, which is our main target, can be visualized in Fig. 6e–g. Paramylon accumulated under aerobic condition during pre-culture seems to decrease with time under anaerobic condition (Fig. 6e). Complementarily, abundance of myristyl myristate (MM1 and MM2), which were not present to begin with at 0 h, slowly starts increasing with passing culture time (Fig. 6f, g). Strong accumulation of wax esters in a localized fashion can be observed starting from 24 h. Interestingly, MM1 distribution (from MCR analysis) is similar to the abundance images obtained using univariate method (Fig. 1e). However, it is also important to note that MCR analysis led to identification of MM2, whose intracellular distribution pattern is quite different from MM1, reiterating the existence of two forms of myristyl myristate. Further clarification of these two forms is left for future studies.

## Conclusions

In summary, we have demonstrated the unique ability of Raman microscopy coupled with MCR analysis to investigate wax ester fermentation and obtain carbon chain length-specific information in single living *Euglena* cells. In the present study, conversion of aerobically accumulated paramylon to MM, a C28 wax ester (C14:0–C14:0), has been successfully visualized. Interestingly, two polymorphic forms of MM with different distribution patterns may have been separated during MCR analysis for the first time in *Euglena* cells. Even though this work focused on specifically identifying MM, we believe that this method can be applied to characterize other metabolites in many different cell types, including but not limited to humans, animals, plants, etc. Moreover, this approach is directly applicable to mutant strains or under other culture conditions. Therefore, our approach is expected to further our understanding of lipid metabolism in *Euglena* and its regulatory apparatus at the cellular level to realize microalgae as an economically viable biofuel feedstock. Moreover, it is clear from the present example that simple univariate analysis, though useful to some extent, is limited by overlapping contributions and that multivariate approach is absolutely necessary to study complex samples of biological origin.

## Methods

### Sample preparation

*Euglena gracilis* SM-ZK, a non-photosynthetic mutant was used in this study. First, *Euglena* was pre-cultured aerobically in Koren–Hutner (KH) medium until stationary phase, diluted 20 times with fresh medium, and cultured aerobically for another 2 days. To perform anaerobic digestion, 1.5 ml of aerobically grown culture was taken in an eppendorf tube of the same volume and sealed with parafilm. All steps were done on a rotary shaker (120 rpm) at 26 °C under dark conditions [12, 29, 30]. For Raman spectroscopic measurements, since *Euglena* are flagellates, 20 µl of culture at each time (0 h, 12 h, 24 h, and 48 h) was put on a concanavalin-A coated glass bottom dish. Then, after standing for about 5 min, a few ml of lukewarm (~ 35 °C) 2% agarose solution was added to further restrict their motion. The glass bottom dish containing *Euglena* cells was then transferred to the microscope as it is for Raman imaging experiment and two cells were measured at each time. All chemical standards were bought either from Sigma-Aldrich or Wako, Japan, and measured using glass bottom dish.

### Raman spectroscopy

Raman spectra were measured using a homemade confocal Raman microspectrometer equipped with a He–Ne Laser (632.8 nm) [16]. The laser beam was introduced into an inverted microscope (Olympus, IX70) and tightly focused onto the sample on the microscope stage using oil immersion objective lens (100×, NA = 1.3). Backscattered light including the inelastically scattered photons was collected by the same objective lens and passed through an edge filter to remove elastic scattering light. In the Raman path, a 50 μm pinhole was set up to achieve confocality before light entered polychromator (Chromex, 250IS). A liquid nitrogen cooled CCD detector operating at − 120 °C (Princeton Instruments, Spec-10) was used to record Raman spectra. The entrance slit width of the polychromator was set to 50 μm and measurements were done using a 600 g/mm grating, resulting in spectral resolution of ~ 4.5 cm^−1^. Lateral and axial resolutions were 300 nm and 3 μm, respectively.

For imaging experiments, a step size of 0.6 μm in *X-* and *Y*-direction was used with the help of a piezo stage (Physik Instrumente). Each euglena cell, being relatively large, took about ~ 40 min to scan the whole cell with an exposure of just 1 s/spectrum. Laser power of 4 mW at the sample point was used for all measurements. An exposure time of 30 s and 60 s was used for measuring several points of lipids and β-1,3-glucan standards, respectively, and averaged. CCD detector and piezo stage were controlled using the LabVIEW software (National Instruments). All measurements were done at room temperature (22 °C).

### Data analysis

Data pre-processing such as dark subtraction, intensity correction (using white light spectrum), and spectral de-noising by singular value decomposition (SVD) analysis were all carried out in IGOR Pro (Wavemetrics). All standard spectra were an average of several points and the fluorescence background was removed by assuming a polynomial baseline.

Raman imaging data from Euglena were analyzed by MCR performed on homemade program written in Python which was used previously [16, 31]. In MCR analysis, matrix approximation sought by a linear combination of desired number of spectral components can be written as follows:

1 $$ A = WH. $$

In this low-rank approximation, *A* is original mapping data of dimension *m* × *n* (*m* denotes number of points per spectrum and *n* denotes the total number of spectra). *W* (*m* × *k* matrix) represents spectral components and rows of *H* (*k* × *n* matrix) represent intensity profile of each spectral component. The parameter *k*, the number of components, can be flexibly decided by referring SVD analysis or a priori estimation. *W* and *H* were iteratively refined using alternating least squares, so that the Frobenius norm ||*A* − *WH*||^2^ is minimized with non-negative constraints *W* ≥ 0 and *H* ≥ 0. A seven-component model (*k* = 7; initialized with six random components and one fixed straight baseline) was constructed. To obtain sparser solutions, L1 penalty term (lasso regression) of *α*^2^ = 0.008 was applied as follows:

2 $$ \left( {W^{\text{T}} W + \alpha^{2} E} \right) \, H = W^{\text{T}} A, $$

where *E* is a *k* × *k* matrix whose elements are all unity. In addition, L2 penalty term (ridge regression) of *β*^2^ = 0.008 was also applied as follows:

3 $$ \left( {HH^{\text{T}} + \beta^{2} I} \right) \, W = HA^{\text{T}} , $$

where *I* is a *k* × *k* identity matrix.

## Data Availability

The authors declare that data supporting the findings in this study are available within the article and raw data can be obtained from corresponding author on reasonable request.

## Abbreviations

MM: myristyl myristate
RS: Raman spectroscopy
MCR: multivariate curve resolution
SVD: singular value decomposition
